# Tick-borne encephalitis in pregnant women: A mini narrative review

**DOI:** 10.1016/j.nmni.2022.101017

**Published:** 2022-08-29

**Authors:** E. Bjonholm, S. Soderholm, O. Stephansson, H.H. Askling

**Affiliations:** 1) Department of Infectious Diseases, Mälarsjukhuset, Eskilstuna, Sweden; 2) Public Health Agency, Sweden; 3) Clinical Epidemiology Division, Department of Medicine, Solna, Karolinska Institutet, Stockholm, Sweden; 4) Department of Women's Health, Karolinska University Hospital, Stockholm, Sweden; 5) Department of Medicine, Division of Infectious Diseases, Karolinska Institutet, Stockholm, Sweden

**Keywords:** Case report, infant, neutralising antibodies, pregnancy, serology, tick-borne encephalitis, tick-borne encephalitis virus, vaccination, vertical transmission, virus, TBE, Tick-borne encephalitis, TBEV, Tick-borne encephalitis virus, PCR, Polymerase chain reaction

## Abstract

Tick-borne encephalitis (TBE) incidence has been increasing in Europe the last decades, but very few cases in pregnant women have been described. We present two cases and describe the serology of both mother and infant at the time of diagnosis and delivery, as well as at months 3, 6, 9, and 12 of follow-up. In both cases, pregnancies and infants developed normally. The mothers had moderate-to severe symptoms of TBE and were positive for IgM and IgG at the time of diagnosis, and throughout the follow up period whilst both infants were PCR- and IgM-negative and positive for IgG during their first months in life. Declining IgG titres were seen in the infants during follow-up until they became negative at the age of nine months. TBE IgG was vertically transmitted in these two cases of infants born to TBE-infected mothers.

## Introduction

Tick-borne encephalitis (TBE) is caused by a flavivirus. The incidence in Europe in 2019 was 0.7/100 000 and the corresponding incidence in Sweden was 3.5/100 000 [1]. Most cases are transmitted by tick bites, but ingestion of unpasteurised dairy products is also a known transmission route of TBEV [2]. There are three described types of TBEV; European (TBE-Eu), Siberian (TBE-Sib) and far Eastern (TBE-FE), where the TBE-Eu is the endemic type in Sweden [3].

TBE can manifests as a range of clinical symptoms; from mild fever to full blown encephalitis and death. The incubation period is 4–28 (median 8) days and the clinical picture for TBE-Eu in adults typically consists of two phases, the first is defined by mild febrile illness followed by an asymptomatic period for about 7–14 days and then a second phase where the symptoms range from mild meningitis to severe encephalitis [4]. The risk of severe disease increases with age, unfortunately corresponding with TBE-vaccination response decreasing in older age. Therefore an extra priming dose is recommended from the age of 50 [5].

TBE is diagnosed by serology with specific IgM and IgG antibodies often present in serum at the time of the first central nervous system (CNS)-symptoms. At this point, TBEV PCR in serum tends to be negative [6] since viremia is only present in the first phase. IgG is present in serum for a long time, and provides lifelong protection against TBE [7]. Both neutralising- and non-neutralising antibodies provide protection against the disease, where the development of neutralising antibodies is considered to be essential for long-life immunity [8,9].

Vaccination is a crucial tool for prevention and has been used effectively for many years in Europe [10]. TBE-vaccines available in Europe are safe and inactivated and can thus be used in both immunosuppressed individuals as well as pregnant women. Natural infection results in higher neutralising antibody titres with less age-dependent decrease compared to passive immunisation after vaccination [6].

Maternal TBEV infection, as described for other Flaviviridae, could also be teratogenic and may also be associated with congenital malformations [[11], [12], [13]].

We present two TBEV-infections in pregnant women including the TBEV serology of both the mothers and the infants during their first year of life. Informed written and verbal consent to provide extra blood-samples, providing the analysis presented in this text, was obtained from the mothers.

## Virological analysis

For clinical diagnostics, specific antibodies of TBEV IgG (ELISA; BioTek 800 TS microplate reader ®) were analysed by Unilabs Skövde and reported as positive or negative. All blood samples taken after diagnosis were analysed at The Public Health Agency of Sweden.

### Patient and infant 1

Analysis for IgM was performed by ELISA by (Immunozym FSME TBE IgM®) in which results are expressed in Vienna units per millilitre (VIUE/mL), according to the manufacturer's instructions. Vienna unit values < 40 VIUE/mL were considered negative, values between 40–126 VIUE/mL as borderline, and values > 126 positive. The IgG antibodies were analysed by ELISA (Ridascreen FSME/TBE IgG®) and expressed in Units per millilitre (U/mL) where values < 100 U/mL were considered negative, values between 100–126 U/mL as borderline, and values > 126 U/mL as positive. Neutralising antibodies were analysed by rapid fluorescent focus inhibition test (RFFIT) results presented in serum dilutions that gives a 50% reduction of infected cells (50% ED50) with a cut off at 5 [14].

### Patient and infant 2

Analysis for IgM was performed by a rapid-test (ReaScan TBE IgM ®) where the result presents as positive or negative with values < 14 considered negative (cut off 8), between 15–30 as borderline and > 30 as positive. IgG was measured by the same method described above (Ridascreen FSME/TBE IgG ®) for the first two samples, and by another ELISA (Serion FSME/TBE IgG®) for the last three samples. The same method was used for NT as above (RFIIT).

**Patient 1:** A 30-year old patient, 31 + 6 weeks pregnant, presented to the labour ward with clinical symptoms and reduced foetal movements. She described five days of headache, neck-stiffness, and aversion to bright lighting, nausea, headache, vomiting, diarrhea, and fever. She was living in a high-endemic area of TBE and reported a tick-bite four weeks prior to start of symptoms. Clinical examination revealed tiredness, low blood pressure of 88/54 mmHg and elevated body temperature of 38^0^C. Cardiotocography (CTG) examination was normal. Abdominal ultrasound revealed a normal amount of amniotic fluid and normal foetal movements. Clinical neurological parameters were normal. Cultures from blood and urine as well as TBE-serology (IgM and IgG) and TMEV PCR were taken. Vital parameters were stable, and the patient was hence allowed to return home during the night. The following morning the clinical status had deteriorated with more tiredness and dizziness. The patient was unable to walk straight without help but had no focal neurological abnormities.

Laboratory results revealed C-reactive protein (CRP) 162 mg/L and leukocyte count of 12 (10ˆ9/L). Cerebrospinal fluid (CSF) showed an elevated total leukocyte count of 133 (10ˆ6/L) dominated by monocytes 114 (10ˆ6/L), protein of 0.7 g/L and a normal glucose index. A meningitis/encephalitis CSF-panel (PCR for *Cytomegalovirus, E. coli, enterovirus, H. influenzae, Herpes simplex 1 and 2, Human herpes virus 6, Cryptococcus, Listeria monocytogenes, Neisseria meningiditis, and Parechovirus)* came back as negative as well as later cultures. The patient was admitted to the infectious disease ward for 4 days and provided with supportive treatment of intravenous fluids, antiemetics and analgesia. She still suffered from headache, but the vomiting and dizziness had improved. Day two post admission, results of the TBE-serology revealed positivity for IgM and IgG (Table 1) as well as TBEV PCR-positivity. The patient had never been vaccinated against TBE. Day 8 post discharge, the patient felt better though still had to lay down most of the day related to dizziness. One month after the first symptoms of TBE, she still felt slight dizziness, but was in much better condition. She was scheduled for a check-up, but prior to that appointment, she gave birth to a healthy infant. Five months post admission the patient confirmed full clinical recovery.

**Table 1:** Presents the results from blood samples taken from patient 1 from the first diagnostic sample and the following nine months post-partum. The results are presented as polymerase chain reaction (PCR) of the TBEV, enzyme-linked immune assay (ELISA) to detect antibodies (IgM and IgG) against TBEV measured in VIEU/mL (Vienna units/mL) with the following reference values for IgM: Neg <40, Pos>126. For IgG the references are: Neg<100 and Pos>126 (units/mL). TBE-NT (neutralising antibodies) with a cut off value of 5. OD-value (optical density) that collaborates with the amount of antibody in the sample. Pos = positive. Neg = negative

	TBE PCR	TBE IgM ELISA	VIEU/mL	OD-value	TBE IgG ELISA	IU/mL	OD-value	TBE NT
Diagnosis of TBE 3 months before delivery	Pos	Pos	>1000	2.35	Pos	1000–3000	1.4	5
Delivery	Neg	Pos	>1000	2.5	Pos	>3000	2.3	5
Month 3	Neg	Pos	478	1.28	Pos	>3000	3.05	40
Month 6	Neg	Pos	214	0.716	Pos	1000–3000	3.16	20
Month 9	Neg	Pos	136	0.456	Pos	1000–3000	2.9	20

The infant was born moderately preterm in week 36 + 5 and required external ventilation for 8 min after birth. Apgar score was 4 + 4 + 6 and admission to the neonatal ward for continuous positive air pressure was required (CPAP) for a few days. This was judged to be a complication of the opioids given to the mother during labour. Already during the first trimester of pregnancy, before onset of TBE, the foetus was diagnosed with a cystic kidney. Apart from the above, the infant was healthy and developed normally during the follow-up period of first year of life. For serological results see Table 1, Table 2.

**Table 2:** Presents the results from blood samples taken from infant 1 from partus to the following nine months post-partum. The results are presented as polymerase chain reaction (PCR) of the TBEV, enzyme-linked immune assay (ELISA) to detect antibodies (IgM and IgG) against TBEV measured in VIEU/ml (Vienna units/ml) with the following reference values for IgM: Neg <40, Pos>126. For IgG the references are: Neg<100 and Pos>126 (units/mL). TBE-NT (neutralising antibodies) with a cut off value of 5. OD-value (optical density) that collaborates with the amount of antibody in the sample. Pos = positive. Neg = negative

Age (months)	TBE PCR	TBE IgM ELISA	VIEU/mL	OD-value	TBE IgG ELISA	IU/mL	OD-value	TBE NT
0	Neg	Neg	<40	0,15	Pos	>3000	2.23	5
3	Neg	Neg	<40	0,1	Pos	1000–3000	1.73	<5
6	Neg	Neg	<40	0,046	Pos	126–200	0.631	<5
9	Neg	Neg	<40	0,047	Neg	<100	0.249	<5

**Patient 2:** In May 2020, a 31-year old woman, who was 26 weeks pregnant, booked an appointment with her general practitioner (GP) because of fever. During the preceding few weeks, she had experienced cold-like symptoms, but the day prior to her visit she developed fever, in the region of 38^0^C, and general muscle- and neck pain along with headache. She reported a tick-bite a few days previously and was concerned about Lyme-disease. General examination by the GP did not reveal any objective signs of Lyme-disease. CRP was taken as well as urine cultures and a nasopharyngeal SARS-CoV-2 PCR, and all came back normal/negative.

Three weeks later, now 29 weeks pregnant, the patient booked a new appointment due to relapse of fever and neck-pain. She had been generally well since the last visit, but three days prior to this visit, had started to feel unwell again with chills, headache, and muscle pain. She was still concerned about a tick-related disease, motivated by her lack of previous TBE-vaccination. Her vitals were stable with a blood pressure 97/61, heart rate 100/min and a body temperature of 37.9°C. CRP, SARS-CoV-2 nasopharyngeal PCR and Lyme serology were all normal/negative. She was sent home in a stable condition but was admitted five days later to the emergency room (ER), afebrile but with a few days' history of severe headache and dizziness. Clinical examination was normal and laboratory check-up revealed CRP 13 (mg/L). The patient was again sent home with the diagnosis viral headache. Four days after the visit to the ER, the GP received a positive TBE-serology IgM and IgG. At this point the patient had no symptoms and no follow-up visits were planned with respect to TBE.

The patient gave birth to a healthy infant week 39 + 5, Apgar 10 + 10 + 10. No complications after birth were reported. For serological results see Table 3, Table 4.

**Table 3:** Presents the results from blood samples taken from patient 2 from the first diagnostic sample and the following nine months post-partum. The results are presented as polymerase chain reaction (PCR) of the TBEV, enzyme-linked immune assay (ELISA) to detect IgG antibodies against TBEV measured in VIEU/mL (Vienna units/mL) with the following reference values for IgM: Neg <40, Pos>126. For IgG the references are: Neg<100 and Pos>126 (units/mL). TBE-NT (neutralising antibodies) with a cut off value at 5. OD-value (optical density) that collaborates with the amount of antibody in the sample. Pos = positive. Neg = negative

	TBE PCR	TBE IgM rapid-test	Value	TBE IgG ELISA	IU/mL	OD-value	TBE NT
TBE-diagnosis 3 months before delivery	Neg	Pos	263	Pos	1000–300	3.7	10–20
Delivery	Neg	Pos	94	Pos	1000–300	2.97	40–80
Month 3	Neg	Pos	58	Pos	3000–1000	3.15	40–80
Month 6	Neg	Pos	43	Pos	3000–1000	1.3	160
Month 9	Neg	Pos	12,6	Pos	3000–1000	2.27	160

**Table 4:** Presents the results from blood samples taken from infant 2 from the first diagnostic sample and the following nine months post-partum. The results are presented as polymerase chain reaction (PCR) of the TBEV, enzyme-linked immune assay (ELISA) to detect IgG antibodies against TBEV measured in VIEU/mL (Vienna units/mL) with the following reference values for IgM: Neg <40, Pos>126. For IgG the references are: Neg<100 and Pos>126 (units/mL). TBE-NT (neutralising antibodies) with a cut off value at 5. OD-value (optical density) that collaborates with the amount of antibody in the sample. Pos = positive. Neg = negative

Age (months)	TBE PCR	TBE IgM rapid-test	TBE IgG ELISA	IU/mL	OD-value	TBE NT
0	Neg	Neg	Pos	1000–300	3.7	5
3	Neg	Neg	Pos	1000–300	2.92	10
6	Neg	Neg	Pos	300–100	1.2	<5
9	Neg	Neg	Neg	Neg	Neg	<5
12	Neg	Neg	Neg	Neg	Neg	<5

## Discussion

TBEV belongs to the family of Flaviviridae and pregnant women and their foetuses could be at higher risk of adverse outcomes. Zika virus is teratogenic and can cause microencephaly. Japanese encephalitis together with dengue fever is associated with higher risk of foetal loss [15]. The state of knowledge regarding TBE in pregnant women is sparse, and only a few cases have been published. Weinmayr et al. presented one case of a pregnant woman in week 21 being admitted due to neurological symptoms including headache and fever. She tested positive for specific antibodies against TBE (IgM and IgG). The pregnancy proceeded well and the newborn showed no signs of infection or altered development after birth. No blood samples were taken, but there were no signs of vertical transmission of the infection on the basis of a completely normal follow up of the infant [16]. Hockicová et al., in 2019 reported a pregnant patient in gestational week 37 who was infected by TBE from food and developed neurological symptoms. She gave birth to a healthy baby with no signs of infection and normal development. The mother recovered fully [17]. Divé et al. presented two women pregnant in week 19 and week 30 (dizygotic twin pregnancy), respectively when diagnosed with TBE. Both women were severely affected, requiring mechanical ventilation in the intensive care unit due to the neurological effects of TBE. They were both diagnosed based on positive serology for TBE in serum as well as CSF and recovered before giving birth. The infants developed normally and showed no signs of intrauterine infection. PCR and IgM were negative for all the infants at birth and after 9 and 11 months. IgG was positive at birth, with a successive decrease over time, becoming negative after 11 and 15 months [18].

In both of our cases, pregnancies and labours proceeded normally. In one of the mothers, PCR for TBEV in blood was negative, corresponding well with the fact that the viremia is not present in the stage of the disease when the patients usually require healthcare. The other mother was PCR TBEV positive which has not been documented before in a pregnant patient as far as we know. Both newborns were TBEV PCR negative in venous blood and this had not been performed in other case reports, where blood from umbilical cord has been used.

Our results indicated transplacental transmission of TBEV IgG with decreasing titres over time, where they no longer could be found after nine months in both cases which is in line with previous findings [19]. Both pregnant women were positive for IgM during the third trimester, but IgM was negative in both newborns, which is expected as IgG is the only antibody class that significantly crosses the placenta [20].

Our results confirm findings from other case-reports showing no evidence of intrauterine transmitted infection, normal pregnancy outcome and normal development of the infants during their first year of life. The added value of this report is 1. A significant contribution to number of cases reported in total, to our knowledge only four women and five infants have been described before [[16], [17], [18]]. 2. Infant samples have been taken from venous blood, which does not have the risk of being mixed with maternal blood as does blood from the umbilical cord 3. Patient 1 was PCR-positive for TBEV during pregnancy, which has not been documented before.

Both families were recommended to vaccinate their children against TBE, after one year of age. The mothers were informed that their immunity against TBE is considered life-long and no further vaccination is recommended, according to existing knowledge of immunity after natural infection [7]. Obstetricians working in the TBE high-endemic areas of Europe should be aware of the disease as a differential diagnosis.

Pregnant women can be vaccinated if highly exposed during pregnancy but to ensure an optimal vaccination response we recommend vaccination of all children and young individuals at risk of TBE, so at the time of future pregnancy they are already protected.

## Transparency declaration

The authors report no conflict of interest.

## Funding

No specific funding linked to this manuscript.

## Author contributions

EB and HHA conceived the idea, SS did the analyses, EB did the literature research, EB and HHA wrote the manuscript, EB, SS HHA and OS reviewed and edited the manuscript. All authors have reviewed and approved the manuscript for publication.

## Key message

TBEV was not vertically transmitted from TBEV-infected patients to their foetuses. Maternal TBEV IgG had vanished nine months after birth in the infants. TBE vaccination is recommended for children born to mothers with TBE-infection during pregnancy, after one year of age.
